# Mentoring as a management practice to retain newly certified professionals in healthcare organisations

**DOI:** 10.1108/JHOM-12-2023-0370

**Published:** 2025-01-27

**Authors:** Roy Liff, Airi Rovio-Johansson

**Affiliations:** Department of Business Administration, University of Gothenburg, Goteborg, Sweden; Department of Pedagogical, Curricular and Professional Studies, Faculty of Social Science, University of Gothenburg, Goteborg, Sweden

**Keywords:** Mentorship, Healthcare professionals, Crisis management, Retention of employees

## Abstract

**Purpose:**

The purpose of this study is to investigate how mentors can convince young, certified, inexperienced employees to remain in a healthcare organisation, and how mentors address “stay or quit” when mentees’ lived experiences reveal feelings of insufficiency as crisis in their daily work. We explore how turnover is affected by the mentors’ and mentees’ discussions within the manager’s domain.

**Design/methodology/approach:**

Within the framework of crisis management, the study employs qualitative content analysis of 21 interview responses from mentors, mentees and managers. The analysis includes mentees’ answers, which are analysed in terms of “weak signals” based on lived experiences and mentors’ and managers’ answers in terms of different capabilities to increase mentees’ wish to remain in the organisation.

**Findings:**

The results show that the deep relationship between the mentee and the mentor is crucial. It is possible for the mentor to detect weak signals from the mentee’s thoughts, doubts and lived experiences. The study extends the understanding of a more subtle mechanism the mentor uses in the close relation to the mentee, alongside the manager. The findings confirm those of previous research concerning improved job satisfaction and self-improvement in the profession.

**Practical implications:**

The findings explain why mentors, as necessary organisational resources, can contribute more successfully than managers to keeping young employees.

**Originality/value:**

The study links the crucial relational mentorship to increased willingness to remain in an organisation among young mentees without career support.

## 1. Introduction

Healthcare organisations constantly face challenges in terms of being attractive employers that provide good working environments for young and newly qualified workers in areas facing employee shortages (European Commission, 2015). Mentoring has positive effects on mentees’ willingness to stay in healthcare organisations (Greene and Puetzer, 2002; Leurer *et al.*, 2007). Research on mentorship shows that behind a growing interest in mentorship is a desire to reduce the risk of younger, inexperienced, well-educated staff choosing to resign, either early or later in their careers (Naim and Lenka, 2016).

Mentoring is a human resource (HR) practice within the healthcare sector (Almeida *et al.*, 2019; Willman *et al.*, 2020). A complementary view regards mentorship as an expression of leadership. This is obvious from more than 40 definitions of mentorship that Haggard *et al.* (2011) identified. In most of these definitions, the mentor provides both career support and psychosocial support. For example, Kram (1983, p. 2) defined mentorship as “a relationship between a young adult and an older, more experienced adult that helps the younger individual to learn to navigate the adult world and working life.” Carmin (1988, p. 10) described the mentoring process as “a complex, interactive process, which occurs between individuals with different levels of experience and expertise that integrates interpersonal or psychological development, career and/or educational development and socialisation functions in the relationship.” These definitions suggest that mentorship assumes co-responsibility for somebody else’s career and experience accumulation. Mentorship is conducted by dialogue and a genuine interest from the mentor to listen to, understand and advise the mentee.

Mentorship programmes can increase three types of support for mentees: (1) psychological and social support, (2) instrumental support and (3) role modelling (Ghosh, 2014; Grocutt *et al.*, 2020; Ullrich *et al.*, 2020). The cited studies have linked the impact of psychosocial support and career support, including technical advice, to the mentees’ willingness to stay. Other studies have suggested that psychosocial support increases mentees’ willingness to remain with the same employer (Scandura and Viator, 1994), because it leads to increased job satisfaction (Lankau and Scandura, 2002), increased commitment to the organisation (Mobley *et al.*, 1994; Weng *et al.*, 2010), increased emotional commitment to their work, and increased participation in the work (Lankau and Scandura, 2002; Stallworth, 2003; Payne and Huffman, 2005), as well as reduced ambiguity in the mentee’s role and fewer role conflicts with colleagues (Scandura and Viator, 1994). Studies have also suggested that mentorship programmes demonstrated positive effects on younger employees’ willingness to stay in the organisation, if more direct career support measures are added to the programme.

The purpose of the present study is to investigate how mentors can convince young, certified, inexperienced employees to remain in a healthcare organisation, and how mentors address the question of “stay or quit” when mentees’ lived experiences reveal feelings of insufficiency as crisis in their daily work. We explore how turnover is affected by the mentors’ and mentees’ discussions within the manager’s domain. Our research question is: How can the relationship – especially more subtle mechanisms of trustfulness – between mentor and mentee explain whether mentorship can affect younger employees’ willingness to stay in an organisation? To answer the research question, it is necessary to take in the viewpoints from both the mentees and the mentors, and also to consider the relationship between the mentee and the manager.

The study was conducted as an interview study. We used crisis management theory, with the concepts of detection, dealing with and making sense of/sensemaking, to interpret results from a qualitative content analysis.

## 2. Previous research

### 2.1 Correlated effects of mentorship on mentees willingness to stay (the general formulation of the research question)

Previous studies have demonstrated that mentees have more successful careers than their unmentored colleagues (e.g. Turban and Dougherty, 1994; Allen *et al.*, 2004; Ramaswami *et al.*, 2010; Ockene *et al.*, 2017). Moreover, support in career development leads to mentees being less interested in changing jobs, and psychosocial support provides a better work environment to facilitate career support (Scandura and Viator, 1994). Training mentees to navigate the organisation supports career development, which Cole (2015) argued makes them better equipped to continue working. Several studies have suggested that well-developed career support, grounded in psychosocial support, needs to be added to affect mentees’ willingness to stay (Scandura and Viator, 1994; Stallworth, 2003; Cole, 2015).

How can subtle mechanisms in the relation between mentor and mentee explain whether mentorship can affect younger employees’ willingness to stay? Some studies have examined these mechanisms as variables in quantitative studies (Turban and Dougherty, 1994) and as the correlation between operationalised variables (Burmeister *et al.*, 2018), with some focusing on the role of the relation between the mentor and the mentee. For example, Turban and Dougherty’s (1994) study of managers (in the role as mentors) and co-workers (in the role as mentees) showed a positive correlation between a mentee’s ability to initiate and develop a relationship with a mentor, and the mentee’s career development and perception of her/his own career as successful.

Straus *et al.*’s (2009) interview study about the mentor–mentee relationship in academic medicine found that good mentorship increases the willingness to remain in the organisation depending on the mentor’s seniority and being approachable accessible, altruistic, patient, honest and understanding. Confidentiality, clear expectations, mutual respect and open communication between the mentee and the mentor were perceived as important.

Similarly, Burmeister *et al*. (2018) emphasised the moderating role of trustworthiness in the relationship between age and knowledge transfer, showing that the mentors’ trustworthiness in the relation benefitted younger employees regarding the perceived motivation to share and receive knowledge. Research on the relationship between the mentor and the mentee has resulted in several positive effects for established experienced mentees in terms of improved job satisfaction and self-improvement in the profession, which makes it likely that mentorship has increased willingness to remain in the organisation (Turban and Dougherty, 1994; Straus *et al.*, 2009; Weng *et al.*, 2010; Burmeister *et al.*, 2018). Psychosocial support leads to increased commitment to the organisation, which leads to increased willingness among the mentees to stay in the organisation (Weng *et al*., 2010).

Greene and Puetzer (2002) found that a structured mentoring programme decreased the number of less experienced nurses who quit because the programme strengthened the relationship between older, experienced nurses and younger, less experienced nurses, promoted teambuilding and helped the less-experienced nurses find their way in the culture and environment. Almeida *et al*.’s (2019) quantitative study found that organising mentoring is an efficient way to allocate organisational resources to facilitate retention of healthcare workers: “The most significant organisational resource was mentoring and guidance on career progression which positively influenced well-being, engagement and job satisfaction” (p. 110).

### 2.2 Subtle mechanisms in the relation between mentors and mentees to influence the mentees’ willingness to stay

To date, no studies have examined how the way a mentor acts directly affects a younger, inexperienced healthcare mentee’s decision process to stay or quit without guidance on career support. Previous studies have not investigated the more subtle mechanism behind a mentee’s decision process regarding whether to stay or quit in healthcare organisations; for example, how the mentor can influence the mentees’ willingness to stay after experiencing a crisis in the daily work situation.

### 2.3 Summary

Since this way of acting as a mentor directly affects the mentee’s decision process, it can be regarded as a kind of leadership. While some previous studies have investigated mentorship as an HR practice, some others have viewed it as a management activity practiced by managers (Harris *et al.*, 2022). However, there is a lack of research on how a mentor can influence the mentee (unlike the manager) to remain in the organisation. The difference between these two actors, with reference to mentorship and the mentees’ will to stay, may be assumed to depend on the relationship between the mentee and the mentor and differences regarding how the mentee perceives the role of a manager vis-à-vis a mentor.

We believe the mentees’ upcoming thoughts about quitting may gradually take the form of a final decision to quit and that it is not appropriate to discuss these thoughts with anyone except for trustworthy individuals. We regard this leadership exercised by mentors as analogous to a crisis management situation for the mentor to handle. It will be a matter of detecting early warning signals from the mentee of thoughts about quitting, which are described in crisis management as weak signals. It will also be a matter of sensemaking of many weak signals, which do not need to indicate such thoughts. The mentor could possibly establish such a confidential relationship with the mentee and influence the mentee’s decision before it is too late to prevent the crisis.

## 3. Theoretical framework

### 3.1 Crisis management

Crisis management theories suggest that crises are processes that develop gradually (Roux- Dufort, 2009; Caj and Yu, 2023). In the first phase, warnings known as “weak signals” are noted and ignored about an incident (Turner, 1978; Roux-Dufort, 2009). Ansoff (1975) asserted that these weak signals appear “early in the life of threat [crises], when the information is vague, and its future course is unclear” (p. 23).

These warnings – “weak signals” – must be identified among various institutional components such as rules, power structure, cognition and value systems (Caj and Yu, 2023). “Power structure will regulate the allocation of responsibility and decision-making power” (Caj and Yu, 2023, p. 244). Weick *et al*. (2005) emphasised that power structure affects the distribution of decision-making power, in this case, between the manager and the mentor.

Such a perception of crisis development emphasises the importance of understanding the ability to handle crisis as an ability to detect weak signals. It is possible to detect weak signals, but difficult to separate them from various signals, which does not indicate a coming crisis. Managers usually have difficulty focusing on information other than the most relevant information and the most relevant issues from the organisation’s perspective, and they therefore disregard peripheral issues and information (Cunha and Chia, 2007).

An organisation needs prerequisites to be able to discern weak signals from irrelevant noise to detect crisis in early stages. Haeckel (2004) and Cai and Yu (2023) argued that organisations need an institutionalised competence for detection, handling and making sense of weak signals and potential coming crises. Making sense of weak signals can be demanding: “Because of their [Crises] low probability, these events defy interpretations and impose severe demands on sensemaking” (Weick, 1988, p. 305).

### 3.2 Mechanisms in exploring crises

In case of immediate *detection* of a mentee’s displeasure with the work situation, the mentor must act, discuss the issue with the mentee and solve the problem as soon as possible. Early detection of the mentee’s dissatisfaction is an important first step in the process of sensemaking (Rovio-Johansson and Liff, 2012). In this step, weak signals emanate from the mentee’s lived experiences (Van Manen, 1990), which are preceded by various daily occurrences that result in interpretations and in a process of sensemaking. If the mentee’s lived experience indicates feelings of insecurity and difficulty coping with daily tasks, the mentor’s actions need to be directed to detecting and handling these weak signals.

In the second step of the process, the mentor must decide *how to deal with* the weak signals. If weak signals concern assignments of tasks in the department, that may seem easier for the mentor to handle than if the mentee has started to doubt his/her own capability to cope with the demands of the profession and of the department. Whatever reason has caused the mentee’s insecurity and feeling of inadequacy, the mentor needs to act immediately.

In a third step, both the mentors and the mentees can, from different perspectives, contribute to the *sensemaking process* of what has happened. In joint discussion between the mentees and the mentors, mentees’ immanent emotions can be shared. Such discussions open the possibility to share and sensemaking of experienced failures, disappointments and successfully organised and solved obstacles at work.

## 4. Setting

The present study was conducted in 2020 in the Region Västra Götaland (RVG), one of Sweden’s 21 elected regions, where healthcare is a main responsibility. RVG is the second-largest region in the country in terms of size, with 1.6 million inhabitants, and Gothenburg is the largest city in the region. RVG provides both emergency and planned treatment in its hospitals (17) and primary healthcare is provided by healthcare centres. The studied healthcare organisations have similar characteristics as the archetypical acute care institution with strong healthcare professionals, as described by Glouberman and Mintzberg (2001).

In RVG, a mentorship programme was developed and implemented in which experienced employees could support younger or inexperienced nurses and physicians in receiving structured guidance from the senior colleague, and thus become confident about providing care and treatment, and would be strengthened in staying and feeling good in their workplace. The implementation engaged approximately 130 mentors in the mentorship programme. The selection started with the unit managers who wanted to participate asking the senior employees (those aged 55 and above) about their interest in being a mentor for 12 months. Younger or less experienced employees at each unit who were interested joined the programme as mentees.

The mentor assignment required the mentor to be physically available to give advice in ongoing situations and participate in discussions, if situations arose, to offer the opportunity for learning afterwards. Special emphasis was placed on the importance of individual, planned conversations with the mentee or in group meetings.

In March 2022, the HR department conducted a follow-up of the staff turnover among the mentees who completed their mentorship programme, regarding the period from 1 January 2020–13 December 2021, for nurses (39), physicians (30), assistant nurses (19), physiotherapists (33) and occupational therapists (2). These were compared with these personnel categories employed in units that were considered equivalent but did not participate in the mentorship programme and for each personnel category in the organisation. The follow-up focused on the percentage of individuals who had resigned at their own request. The following results were obtained: nurses (10% with a mentor versus 18% without), doctors (none versus 7%), assistant nurses (5% versus 15%), physiotherapists (3% versus 19%), occupational therapists (none versus 3%). Similar results were found in the comparison for each staff category for the entire organisation. The report on the comparison revealed that the mentorship programme has led to the mentees remaining in VGR to a greater extent than the employees who did not participate in the mentorship programme (Bjarnemark, 2022).

## 5. Method

### 5.1 Design

The study was conducted as a case study (Creswell, 2013), where the studied unit was an HR-initiated mentorship programme that was rolled out to different categories of healthcare professionals in different healthcare settings in the region. This case study aimed to study real-life situations with the intention of achieving an in-depth understanding of interviewees’ context-dependent lived experiences (Creswell, 2013). In the present study, this concerned how mentors and managers can convince young, certified, inexperienced employees to remain in healthcare organisations.

This is a single-case study, which has an advantage compared to a multi-case design in that it is easier to focus on the more implicit, subtle mechanisms, such as relationships between actors in different situations (Dyer and Wilkins, 1991). Within the same context studied in a single case, a comparative approach can be achieved by contrasting different aspects and factors that impact on the phenomenon (Dyer and Wilkins, 1991); in the present study, this was done by comparing the mentees’ relation to mentors with their relation to managers.

### 5.2 Sampling, participants and their background

Stratified purposeful sampling was used among the total number of participants, as it is a method that “illustrates characteristics of particular subgroups of interest” (Patton, 1990, p. 174). The study included 21 interviews: seven with mentees, five with mentors and nine with managers. The mentees were aged 25–30 and had not more than two years of experience after their examination. The mentors were aged 60–63 and have had lifelong careers. The managers were in the age range between those of the mentees and mentors and held stable positions in the organisation. The mentors and mentees represented three professional groups (physicians, nurses and assistant nurses) from both surgical and medical specialties and from both hospital units and open health care units. Accordingly, the participants were strategically selected to represent the professional categories that participated in the mentorship programme, such as participants of medical and surgical specialties, and personnel from inpatient and outpatient care and different care units across the region (see Table 1). After achieving a balance in gender distribution, the aim was to maximise the variation in the background variables (Bell *et al*., 2019).

**Table 1:** Participants’ roles and departments

Role in research project	Rehabilitation dept.	Medicine dept.	Psychiatry dept.	Health care Centre	Radio-logy dept.	Total
Mentor	1	2	2			5
Manager	3	2	2	1	1	9
Mentee	3	2	2			7
Total	7	6	6	1	1	21

**Source(s):** Authors’ own work

### 5.3 Interviews and data collections

For this qualitative research, the method of semi-structured interviews was chosen (Bell *et al.*, 2019). Such interviews are used for gathering information on a specific topic (Rubin and Rubin, 2012; Bell *et al.*, 2019). The interview guide is used as a general frame for the interview as the questions are listed specifically so that each topic is covered adequately. The interview guide started with general questions on the respondents’ background, occupation, role and years within the organisation and in the mentorship programme. This was followed by questions about what meaning they attribute to mentorship; what needs they think the mentees have; reasons why younger, less experienced employees quit; what effects can be expected and what conditions exist for successful mentorship in their own organisation. Furthermore, the interview questions to the mentees addressed how the mentor programme had changed the work environment and their own competence, the significance of the mentor programme for the desire to remain in the workplace, and whether the mentor programme could have been set up differently. The interviews with the mentees covered the following questions:

1. During your time as a mentee, have you thought about changing jobs or changing workplaces? Have you considered quitting where you are now? Has that aspect been discussed with your mentor?
2. Hypothetical questions: Do you now experience self-doubt or question whether you are the right person for the profession? If so, with whom would you raise that aspect and discuss it in the workplace?

The interviews with the managers covered the following questions:

1. Could anything be detrimental to you in your business if the mentor starts setting out paths for the mentee that lead to moving on? Do you see any competing effect in the mentor addressing the mentee’s career development thoughts in relation to what you do in the development conversations with the mentee, your co-worker, in this case?
2. Do you feel it is easier for the mentee to discuss his/her future also in terms of “I intend to quit” with a mentor or with you as a manager in development talks?

The interviews with the mentors covered the following questions:

1. What do you think you can contribute in relation to the mentee? What are you expecting to achieve with your effort?
2. What is the purpose of the effort? Is it about transferring experiences? Is it about improving the working environment for the mentees?

The interviews were accomplished by two researchers with extensive experience of conducting semi-structured in-depth interviews (Brinkmann and Kvale, 2018). All interviews were conducted via TeamLink. This process was chosen based on the recent COVID-19 pandemic, along with the logistical benefits from conducting the interviews remotely. All interviews lasted approximately 30–45 min and were audio-recorded and transcribed.

### 5.4 Data management and data analysis

The study followed Yin’s (2016) five steps for data analysis. In the *first* step, the texts of the transcribed 21 interviews were assembled, condensed and organised after the answers to each question, in three categories – respondents, mentors, mentees and managers – labelled by who had given the answer. In *the second step,* the researchers read the collected interview answers iteratively to gain a deeper understanding of managers’, mentors’ and mentees’ answers, and the main impressions of the answers were noted to obtain a first impression of what the respondents thought, question by question (Strauss and Corbin, 1990).

*The third* s*tep* included a deeper qualitative analysis of the text of the 21 interviews ordered in the first steps. In applying a *qualitative content analysis*, there is a need for definitions of *context*, *units of analysis, analytical rules*, *meaning units* (codes) and *categories* (Graneheim and Lundman, 2004; Mayring, 2022). The focus of analysis was on the mentees’ statements (*unit of analysis*) in the interviews, interpreted as their lived experiences (Van Manen, 1990) in the *context* of healthcare work, their skills at work, and how well they fit into the profession. *Analytic rules* refer to the iterative reading of interview answers and the line-by-line analysis of selected quotations, which illustrates the mentees’ different experiences. As mentioned above, *lived experiences* (Van Manen, 1990) refer to how the mentees’ experience and understand the healthcare work as real and meaningful work (Denzin and Lincoln, 2018; Mayring, 2022). The *meaning units* are *codes* in interviews, which were jointly identified and discussed by the researchers. After that, categories were jointly constituted and discussed to achieve consensus in the analysis. Eventually, the *categories* attained were as follows: (1) *experiences of insufficiency,* (2) *missing professional skills,* and (3) *quitting or staying.*

In *the fourth step,* we analysed the re-assembled material according to these categories based on our theoretical framework (see Section 3.2). Mentors’ and managers’ analysis, as understood by the researchers, are interpretations of the categories of *weak signals* understood as mentees’ lived experiences in terms of *detection*, *dealing with* and *making sense of/sensemaking* (Table 1). We employed these concepts as precepts for the further content analysis (Hsieh and Shannon, 2005). Finally, in *Step 5,* we selected the quotations that were the most illustrative of the respondents’ opinions and their analyses of their opinions.

### 5.5 Credibility of data and consistency of results

In this case study, evidence to support the credibility (internal validity) of collected data is based on applied rigorous techniques for collecting data, qualitative content analysis, and the abilities of two experienced researchers (Denzin and Lincoln, 2018; Patton, 1990), who conducted a deep holistic analysis of interviewees’ responses in the interviews. External validity and the generalisability of results will be discussed together with the limitations of the study.

In social science research, human behaviour is never static, nor are lived experiences. “Replication of a qualitative study will not yield the same results, but this does not discredit the results of any particular study; there can be numerous interpretations of the same data” (Merriam and Associates, 2002, p. 27).

To ensure the trustworthiness and consistency of results, both researchers independently analysed and codified the answers given by the interviewees (interrater reliability/peer examination). Two answers showed discrepancies between the researchers’ codifications. After thorough discussions, the discrepancies were ironed out and consensus was achieved in assessments. Interrater reliability was estimated to be 0.90% and was accepted as very good by both researchers (Denzin and Lincoln, 2018).

### 5.6 Ethical considerations

The data collected in the interviews did not include any personal or sensitive data concerning mentees, mentors or managers; therefore, the study did not need to be tested and approved by the National Ethical authority.

The participants in the study have, through informed consent (Swedish Research Council, 2017), agreed to ethical considerations concerning the aim of the study, anonymity, confidentiality and integrity for research participants before deciding to participate in the study. At the start of the interviews, the interviewer reminded the interviewees of the aim of the study, and that they were participating voluntarily and could stop and finish the interview whenever they wanted. All collected data are confidential and treated according to the European Union Regulation (General Data Protection Regulation, GDPR, 2016).

## 6. Findings and analysis

The study identified two answers to the research question of why the mentorship programme can be expected to prevent younger, less experienced employees from leaving prematurely and unnecessarily. First, based on the interview study, and supported by the internal analysis of turnover data, it is likely that the significant improvements in increased security in daily work, experience of support at work, and experience of increased competence have a positive impact on the mentees’ willingness to remain in their workplace. Second, relationship-building between the mentor and mentee is imperative for the mentees’ decision to stay.

The first answer to the research question is given from the perspective of the mentees, mentors and managers. The perspective of the mentees is described as their overall impressions of the mentor programme (6.1). The perspectives of the mentors and managers were investigated from their responses to the mentees’ experiences of (1) insufficiency, (2) missing professional skills, and (3) quitting or staying, which are the categories emanating from the qualitative content analysis. The findings in these categories were interpreted by the concepts in the theoretical framework of weak *signals* in terms of *detection*, *dealing with* and *making sense of/sensemaking*. Subtitles 6.2–6.4 are combinations of these categories and the theoretical concepts as a result of the interview answers.

### 6.1 Overall impression of the mentorship programme’s effects from the mentees’ perspective

The mentorship programme has focused on knowledge transfer, such as supervision. Hence, the mentees have mainly wanted support to develop their clinical competence and have not specifically requested reflective conversations with their mentor. This relationship can be seen as natural in the first stage of a mentorship for younger and newly hired employees. The mentorship programme presupposes a more systematic and structured approach that may eventually lead to the mentees wishing to see more of a whole in their work, which will then demand reflective conversations, to a greater extent. However, the focus has not related to the mentees’ development process, either in managerial careers or in a skills development ladder.

For physicians, the mentorship programme is similar to supervision, with gradually freer forms and an increasing element of what the mentee needs regarding knowledge transfer. For mentees with nursing professions (nurses, assistant nurses and mental health nurses), a role model from previous supervision methods does not seem to be as governing, which may have affected the view of mentorship as being different for physicians and mentees in nursing professions.

The mentorship programme is linked to strategies that the mentors and their managers consider necessary in order to develop competence within the activities and with processes that are accepted for competence transfer. The idea of ​​a mentor programme is clearly viewed as positive; that is, the individual’s offer to be a mentor has been received positively throughout. One explanation may be that the participating units have had the freedom to design the mentorship based on their conditions and needs.

### 6.2 Mentors’ and managers’ detection of mentees’ lack of experiences of insufficiency

The interviews with the mentees indicated that they may feel inadequate in their professional practice, due to a lack of experience and to overly high demand, which can lead to doubts about wanting to continue.

Interview (I) 9. “If you get to share your insecurities and your thoughts with a more experienced person, you don’t carry with you this feeling of “Oh, I’m inadequate”. Then you become more secure in your job; you are a little more secure with the results. It can ease the feeling of insecurity that easily can create guilt for everything you didn’t do, what you should or could. It has a lot to do with one’s mental health, well-being at work. That stuff weighs very, very heavily.” (Mentor/Occupational therapist, rehab clinic)

I 7. “There is a more reflective element about her way of functioning as a nurse. You can tell how they feel inside the soul. Don’t go home feeling burnt out and not happy with the day. Inadequacy is a concept that comes up often in our profession.” (Mentor/nurse, rehabilitation medicine unit)

The mentor contributes by confirming the mentee’s ability, which has significantly reduced the mentee’s doubts and feelings of inadequacy. The mentor helps the mentee relate her/his capability to what can be regarded as normal and good enough, thus reducing the mentee’s feeling of inadequacy in her/his professional role.

The manager may think the mentee’s career support will come close to the manager’s role but also realise the mentor can have different conversations than the manager.

I 13. “Conversations about the mentee’s career paths are common [for] me. But you can have that as a mentor as well. You certainly talk differently to a colleague who is a mentor than to your manager.” (Unit manager, rehab clinic)

I 19. “Anything that has to do with insecurity might not be something you want to show the manager. How do you bring it up? I think a mentor can better catch such signals early on. I think many people think they are in the manager’s hands if they start to flag that they don’t want to stay, that they can’t attend training and that they don’t get a salary increase. I also think it is important that certain things do not leave the mentor’s room. So, a mentee must also be able to feel that they can talk to the mentor about everything. If there is something they want to pass on to me, then they do it together.” (Nursing unit manager, psychiatry)

Overall, managers believe it is easier for a mentee to raise their insecurities with their mentor than with their manager. It may also be easier for the mentee to express early thoughts about leaving to the mentor. This is because conversations with the mentor do not risk leading to uncontrolled consequences for the mentee, which could be the case with conversations with the manager about, for example, thoughts of quitting. Thus, the mentor is considered to have better conditions than the manager to catch early signals of concern that the mentee feels in his/her professional practice and in their confidence.

### 6.3 Mentors and managers dealing with mentees’ missing professional skills

The mentor can help the mentee understand what behavioural aspects he or she needs to change to help them to stay at the workplace.

I 11. “I think this programme gives me room to adapt my contribution based on the needs of the mentee regarding social aspects, if that is what is needed.” (Mentor/nurse, psychiatry)

I 10. “Many nurses today are quite good at healthcare technology. It is instead about guidance regarding how they should prioritise. These are normal, small priorities during today’s work.” (Mentor/nurse, rehabilitation medicine unit)

The mentee can learn pragmatic methods that are good enough within reasonable levels of quality aspirations. Another view is that the mentor can give the mentee a complementary view that the manager may provide more long-term development issues for the mentee.

I 18. “It depends on what kind of relationship you have with the employees regarding how much you, as a manager, find out beforehand. It may be that you have gained trust in your mentor more than in the manager that you dare to open more to the mentor. The mentor can help clarify a collegial norm.” (Manager/head of care centre)

I 15. “So, the mentees’ career plans are between me and the employees as well, we have development plans. But I have nothing against them [the mentor and the mentee] discussing such things. Then, if it’s a cost issue or something to do with staffing, it must go through me, anyway. But that they argue and discuss and bring things up, absolutely! That’s great.” (Manager/nursing unit manager, psychiatry)

The mentor may regard the mentee’s career support as complementary to the manager’s career support, perhaps in a sequence where the mentor starts such conversations, which the manager later materialises.

### 6.4 Mentors’ and managers’ sensemaking and handling of mentees’ decision to quit or stay

A mentor who has a trusting relationship with the mentee can ask the mentee reflective questions without the expectation that the discussion will end up with the mentee wanting to quit. This conversational practice may reveal all the reasons for self-doubt and make it possible for the mentor to analyse all the relevant arguments together with the mentee.

I 8. “He has wondered: ‘Should I stay in this organisation? Do I need to find my way back to something else where I can keep my previous role as a nurse?’ We have reflected on this work being different and therefore, you must find a professional role in it as well. And at the same time, you must also honestly say that sometimes it is also good to decide not to stay.” (Mentor/nurse, psychiatry)

One view is that there is a competitive relationship with what the manager raises with the mentee in development talks and that there is a risk that the mentee will receive advice that leads to him/her leaving the unit.

I 12. “If you have a mentor who speaks for the mentee to become a doctoral student, then the risk is that you lose the employee. It can be positive for the profession, but for us it would be a disadvantage.” (Unit manager, rehab clinic)

I 16. “Yes, I think it’s easier with a mentor than to bring it up with me. So, they can have a different discussion than we can have.” (Unit manager, rehab clinic)

The mentor may be able to identify weak signals from mentees who are thinking of quitting and may therefore be able to reduce the mentee’s doubts at an early stage, while conversations with the manager may occur in a later more or less definite stage of the mentee’s decision process.

I 17. “The mentor could be someone with whom you can raise concerns about quitting without any consequences. Therefore, the mentor would be in a better position to discover this early than the manager if the mentee is considering quitting. I think that would certainly be a good role to fill.” (Manager/head, Radiology)

Serious discussions with the manager about quitting may have consequences for the mentee’s future position in the organisation, unlike a conversation with the mentor. The mentor may discuss the mentee’s thoughts about quitting and be able to reduce the mentee’s doubts at an early stage, while conversations with the manager may occur later.

### 6.5 Summary: the intention is to prevent younger, less experienced employees from leaving

First answer to the research question:

The first answer to the research question is summarised in Table 2.

**Table 2:** Mentors’ and managers’ analysis of “weak signals” (WS) as mentees’ lived experiences

1. Mentors’ detection of mentee’s WS	1. Managers’ detection of mentee’s WS
Experiences of inadequacy Mental health/well-being at work Satisfied in work and profession	Mentors can catch weak signals earlier You don’t show insecurity to manager Decisive talk with mentor, not manager
2. Mentors dealing with mentees’ WS	2. Managers dealing with mentee’s WS
Develop a certain attitude to work Guidance on how to prioritise in work Mentor adapts to mentee’s needs	Depends on the relationship More trust in mentor than manager Grow skills in development plans
3. Mentor’s sensemaking of WS	3. Manager’s sensemaking of WS
The choice: to stay or quit Develop professional role Support is decisive	Talk about quitting but not with managers Mentor has different discussion - Mentor understands differently

**Source(s):** Authors’ own work

With the help of the theoretical concepts, Table 2 summarises how mentors and managers detect weak signals from the mentees of inadequacy and insecurity, and how the mentors and managers deal with these weak signals and make sense of them to influence the mentees to stay. The table demonstrates the difference between the mentors and managers’ ability to influence the mentees to stay. In the first line in Table 2, the managers point out that the mentors, rather than the managers, are the first to be informed when the mentees’ lived experiences indicate that they realise their lack of competence for the profession. In the second line, the mentors inform the mentees that the profession demands a certain professional attitude and ability to prioritise, while the managers assert that the mentees will reach necessary skills in the development programs offered. Finally, in the third line, the difficult decision that the mentees must make – to “stay or quit” – requires support primarily from the mentors, according to the managers.

Second answer to the research question:

The second explanation of why mentorship may contribute to a younger employee’s willingness to stay is that relationship-building between the mentor and mentee has allowed the mentee to raise thoughts about quitting at an early stage without facing decisive and definitive consequences, as such conversations with a manager might have. Therefore, the mentor may help reduce or solve the problem without it becoming known in the organisation that the mentee is considering leaving the workplace.

Another conclusion is that, by listening to the mentee, the mentor can mitigate the mentee’s feeling of dissatisfaction, despite factors that the mentor cannot influence, such as salary, work pace and work content. The mentor explains that the mentee’s perceived difficulties are normal for a young professional.

## 7. Discussion

The mentorship works as a deliberative management practice, which is a method of deliberating on what to do in different situations, building practical identity, helping the mentee bridge the gap between “thought” and “action”, and building social capital in the form of trust and goodwill in the joint evaluation of actions. We expand on Burmeister *et al.*’s (2018) notion of how trustworthiness positively mediates knowledge transfer with the conclusion that the close relationship between the mentor and mentee influences the mentee’s overall assessment of wanting to stay within the organisation.

The effects on the mentee’s willingness to stay depend on factors such as:

1. The nature of the dialogue within the relationship
2. The use of mentorship in an early stage of employment
3. Opportunity to detect and deal with weak signals before sensemaking
4. Ways for mentees to normalise what they perceive as specifically weak regarding their level of development
5. Normalising the mentee’s lived experience regarding what is a normal experience in the chosen profession.

The results of the current study support several studies that have argued that the relationship between the mentor and the mentee is crucial to improved job satisfaction and self-improvement in the profession, and that this leads to a lower willingness to quit (Straus *et al.*, 2009; Weng *et al.*, 2010; Greene and Puetzer, 2002; Almeida *et al.*, 2019). However, the present study extends the understanding of the more subtle mechanism. Thanks to a close relation to the mentee and a position alongside the manager, the mentor could get early information from the mentee’s weak signals; specifically, lived experiences about the mentee’s thoughts about whether to stay or quit. For the mentor, a necessary prerequisite is to detect, understand and intervene in the mentee’s decision process, which the manager cannot do due to the mentee’s needs to not fear consequences and to get into a definite position, by revealing thoughts of doubts about their competence and willingness to stay in the organisation.

Our findings also confirm earlier research that mentorship programmes can affect the mentee’s job satisfaction and can be assumed to have preventive and health-promoting effects on the work environment for both new and older employees (Scandura and Viator, 1994; Seibert *et al.*, 2001; Lankau and Scandura, 2002; Almeida *et al.*, 2019; Côté *et al.*, 2019). Following Scandura and Viator (1994) and Lankau and Scandura (2002), we show that psychosocial support increases mentees’ willingness to remain within the organisation since the mentorship programme increases the mentee’s job satisfaction and reduces ambiguity in the mentee’s role.

In line with Côté *et al.* (2019), the present study also shows that a mentorship programme helps mentees handle the stress that arises during the first years in the healthcare profession. We also confirm Seibert, Kraimer and Liden’s (2001) results that the transition for mentees of going from novice to expert is highly stressful. The mentor programme supports the mentees during this transition, both in handling difficult patient situations and increasing their ability to collaborate with colleagues.

This study shows that the most important factor in the mentee’s decision is not the specific composition in the mentorship of psychosocial support, of knowledge transfer or direct career support, but the direct influence in the mentee’s decision-making process. This becomes possible if the mentor dedicates his/her support to what the mentee perceives as his/her primary need for support; in this case, to develop professional, social and technical skills based on mentee’s lived experiences.

## 8. Conclusion

The purpose of this study was to investigate how mentors can convince young, certified, inexperienced employees to remain in a healthcare organisation, and how mentors address “stay or quit” when mentees’ lived experiences reveal feelings of insufficiency as crisis in their daily work. This was studied by interviewing mentors, mentees and managers and analysed using crisis management theory.

This study has three main conclusions. First, technical skills are of great importance for the mentee’s willingness to stay rather than a better work environment in general. Second, the confidential relationship that is created between the mentor and mentee allows for conversations about the mentee’s doubts/willingness to stay. Mentorship could serve as a deliberative management practice regarding what to do in difficult situations. That means helping the mentee bridge the gap between knowledge and action and building social capital in the form of trust between mentors and mentees. Third, mentoring can be seen as an informal form of leadership. An organisation’s ability to deal with the fact that younger people quit too early and unnecessarily depends on the mentors’ abilities to cope with the informal conversations, where mentees reveal their incompetencies, doubts and lack of self-confidences and their thoughts about quitting. Without this informal, confidential leadership that mentorship can contain and represent, an organisation based on formal leadership alone cannot reduce staff turnover among younger employees.

The above points are the most important ones and represent the main contribution of this study. Mentoring works as a confidential relationship and a mechanism for the mentee’s willingness to stay within the organisation, even without direct career support measures, which would further strengthen the mentee’s willingness to stay. In the current professional healthcare culture, the missing career support in the study can be explained by the fact that it is regarded as a foreign element.

### 8.1 Practical implications

In an expanded notion of what mentorship can and should mean, focused on the intention to influence the mentees’ willingness to stay, it is important that the mentors understand that the prerequisite for a successful mentorship is deep trust between the mentor and the mentee. It is essential that the mentor conversations include in-depth reflective conversations about the mentees’ development and future development in order to understand what lived experience is for the mentee. In the current study, interview answers indicated that deep trust existed between the mentor and the mentee when talking about mentees’ lived experiences. It was easier for the mentees to talk about plans to quit with mentors than with managers. Mentorship also includes more strategic issues concerning the mentees’ career paths within the organisation. Besides, the mentor must have a clear idea of what should be included in the mentor assignment; for instance, certain conversations may require a certain technique. Younger employees may hide their insecurities and thoughts about wanting to quit; a mentor should have conversations with these employees about such aspects that cannot be assumed to be explicit but may still be decisive for a decision to quit.

### 8.2 Implications for methodology and theoretical framework

The selected theoretical framework that made it possible to detect and address “weak signals” of potential turnover had served the purpose of studying the subtle and confidential relationship between mentor and mentee.

### 8.3 Limitations of the study

The study is limited to just one healthcare region and comprises a limited selection of interviewed mentors and mentees. Since the focus is not on how common a specific opinion is, but on the sample’s *opinion*, we know that a wide variety of backgrounds, such as hospital care, open care, and various special medical fields and mentors’ positions in the organisation variables, affect the possibility to make analytical generalisations. The conclusions are generalisable not only to the studied region. The kind of relationships among managers, employees, and senior members of the staff who are willing to take on mentorship can be similar to the case in other public organisations, which leads us to believe that the conclusion of this case study in a healthcare organisation is highly comparable to different public organisations. However, a limitation of the study is that we studied subtle relationships between mentors and mentees by interviewing the actors on one occasion, rather than participating as observers in their conversations.

### 8.4 Future research

Finally, the conclusion of this case study in a healthcare organisation is transferable to other similar public organisations, so further studies in similar contexts would be of interest. More thorough studies of the confidential relationship between the mentor and mentee, including observations of their interaction, would be interesting and could provide a more detailed and deeper understanding of the significance of enhancing the mentee’s technical skills. Furthermore, studies about the reciprocal effects of mentorship on the mentors and their willingness to prolong their length of service would be of interest.

## References

[ref001] Allen, T.D., Eby, L.T., Poteet, M.L., Lentz, E. and Lima, L. (2004), “Career benefits associated with mentoring for proteges: a meta-analysis”, Journal of Applied Psychology, Vol. 89 No. 1, pp. 127-136, doi: 10.1037/0021-9010.89.1.127. 14769125

[ref002] Almeida, S., Fernando, M., Munoz, A. and Cartwright, S. (2019), “Retaining health carers: the role of personal and organisation job resources”, Journal of Organizational Effectiveness: People and Performance, Vol. 6 No. 2, pp. 98-113, doi: 10.1108/joepp-06-2018-0036.

[ref003] Ansoff, H.I. (1975), “Managing strategic surprise by response to weak signals”, California Management Review, Vol. 18 No. 2, pp. 21-33, doi: 10.2307/41164635.

[ref004] Bell, E., Bryman, A. and Harley, B. (2019), Business Research Methods, 5th ed., Oxford University Press, Oxford.

[ref005] Bjarnemark, N. (2022), “Halvtidsrapport – Mentorskap i senior kompetens Diarienummer: RS 2018-06570”, Västra Götalandsregionen.

[ref006] Brinkmann, S. and Kvale, S. (2018), Doing Interviews, 2nd ed, Sage Publications, Thousand Oaks, CA.

[ref007] Burmeister, A., Fasbender, U. and Deller, J. (2018), “Being perceived as a knowledge sender or knowledge receiver: a multi study investigation of the effect of age on knowledge transfer”, Journal of Occupational and Organizational Psychology, Vol. 91 No. 3, pp. 518-545, doi: 10.1111/joop.12208.

[ref008] Caj, C. and Yu, Z. (2023), “Through the mist: how institution-knowledge bricoleurs make sense of a crisis”, International Public Management Journal, Vol. 26 No. 2, pp. 240-257, doi: 10.1080/10967494.2022.2155739.

[ref009] Carmin, C.N. (1988), “Issues on research on mentoring: definitional and methodological”, International Journal of Mentoring, Vol. 2 No. 2, pp. 9-13.

[ref010] Cole, G. (2015), “The value of mentoring”, Development and Learning in Organizations, Vol. 29 No. 4, pp. 22-24, doi: 10.1108/dlo-04-2015-0039.

[ref011] Côté, L., Deschênes, D., Hudon, E., Galarneau, S. and Bolduc, G. (2019), “Quebec College of family physicians' new formal mentorship program”, Canadian Family Physician, Vol. 65 No. 11, pp. 481-486. 31722929 PMC6853349

[ref012] Creswell, J.W. (2013), Qualitative Inquiry and Research Design: Choosing Among Five Approaches, 2nd ed., Sage Publications, Thousand Oaks, CA.

[ref013] Cunha, M. and Chia, R. (2007), “Using teams to avoid peripheral blindness”, Long Range Planning, Vol. 40 No. 6, pp. 559-573, doi: 10.1016/j.lrp.2007.08.004.

[ref014] Denzin, N.K. and Lincoln, Y.S. (2018), Handbook of Qualitative Research, 5th ed., Sage Publications, Los Angeles.

[ref015] Dyer, G. and Wilkins, A.L. (1991), “Better stories, not better constructs, to generate better theory: a rejoinder to Eisenhardt”, Academy of Management Review, Vol. 15 No. 3, pp. 613-619, doi: 10.2307/258920.

[ref016] European Commission (2015), Recruitment and Retention of the Health Workforce in Europe, Public Health Management Association, Brussels.

[ref017] European Union (EU) (2016), General Data Protection Regulation, Regulation (EU) 2016/679, European Parliament and Council, EU.

[ref018] Ghosh, R. (2014), “Antecedents of mentoring support: a meta-analysis of individual, relational, and structural or organizational factors”, Journal of Vocational Behavior, Vol. 84 No. 3, pp. 367-384, doi: 10.1016/j.jvb.2014.02.009.

[ref019] Glouberman, S. and Mintzberg, H. (2001), “Managing the care of health and the cure disease – Part I: differentiation”, Health Care Management Review, Vol. 26 No. 1, pp. 56-69, doi: 10.1097/00004010-200101000-00006. 11233354

[ref020] Graneheim, U.H. and Lundman, B. (2004), “Qualitative content analysis in nursing research: concepts, procedures and measures to achieve trustworthiness”, Nurse Education Today, Vol. 24 No. 2, pp. 105-112, doi: 10.1016/j.nedt.2003.10.001. 14769454

[ref021] Greene, M.T. and Puetzer, M. (2002), “The value of mentoring: a strategic approach to retention and recruitment”, Journal of Nursing Care Quality, Vol. 17 No. 1, pp. 63-70, doi: 10.1097/00001786-200210000-00008. 12369749

[ref022] Grocutt, A., Gulseren, D., Weatherhead, J. and Turner, N. (2020), “Can mentorship programmes develop leadership?”, Human Resource Development International, Vol. 25 No. 4, pp. 404-414, doi: 10.1080/13678868.2020.1850090.

[ref023] Haeckel, S.H. (2004), “Peripheral vision: sensing and acting on weak signals making meaning out of apparent noise: the need for a new managerial framework”, Long Range Planning, Vol. 37 No. 2, pp. 181-189, doi: 10.1016/s0024-6301(04)00030-5.

[ref024] Haggard, D.L., Dougherty, T.W., Turban, D.B. and Wilbanks, J.E. (2011), “Who is a mentor? A review of evolving definitions and implications for research”, Journal of Management, Vol. 37 No. 1, pp. 280-304, doi: 10.1177/0149206310386227.

[ref025] Harris, C.M., Brown, L.W. and Pattie, M.W. (2022), “You are drafted: the role of employee and manager human capital on employee career advancement”, Journal of Organizational Effectiveness: People and Performance, Vol. 9 No. 3, pp. 506-523, doi: 10.1108/joepp-07-2021-0189.

[ref026] Hsieh, H.F. and Shannon, S.E. (2005), “Three approaches to qualitative content analysis”, Qualitative Health Research, Vol. 15 No. 9, pp. 1277-1288, doi: 10.1177/1049732305276687. 16204405

[ref027] Kram, K.E. (1983), “Phases of the mentor relationship”, Academy of Management Journal, Vol. 26 No. 4, pp. 608-625, doi: 10.2307/255910.

[ref028] Lankau, M.J. and Scandura, T.A. (2002), “An investigation of personal learning in mentoring relationships: content, antecedents, and consequences”, Academy of Management Journal, Vol. 45 No. 4, pp. 770-790, doi: 10.5465/3069311.

[ref029] Leurer, M.D., Donnelly, G. and Domm, E. (2007), “Nurse retention strategies: advice from experienced registered nurses”, Journal of Health Organization and Management, Vol. 21 No. 3, pp. 307-319, doi: 10.1108/14777260710751762. 17713190

[ref030] Mayring, P. (2022), Qualitative Content Analysis. A Step-By Step Guide, 5th ed., Sage, Los Angeles.

[ref031] Merriam, S.B. and Associates (2002), Qualitative Research in Practice. Examples for Discussion and Analysis, Jossey-Bass, San Francisco, CA.

[ref032] Mobley, G.M., Jaret, C., Marsh, K. and Lim, Y.Y. (1994), “Mentoring, job satisfaction, gender, and the legal profession”, Sex Roles, Vol. 31 Nos 1-2, pp. 79-98, doi: 10.1007/bf01560278.

[ref033] Naim, M.F. and Lenka, U. (2016), “Mentoring as an HR intervention to engage Gen Y employees”, People: International Journal of Social Sciences, Vol. 2 No. 1, pp. 1697-1715, doi: 10.20319/pijss.2016.s21.16971715.

[ref034] Ockene, J.K., Milner, R.J., Thorndyke, L.E., Congdon, J. and Cain, J.M. (2017), “Peers for promotion: achieving academic advancement through facilitated peer mentoring”, The Journal of Faculty Development, Vol. 31 No. 3, pp. 5-13.

[ref035] Patton, M.Q. (1990), Qualitative Evaluation and Research Methods, 2nd ed, Sage Publications, Newbury Park, CA.

[ref036] Payne, S.C. and Huffman, A.H. (2005), “A longitudinal examination of the influence of mentoring on organizational commitment and turnover”, Academy of Management Journal, Vol. 48 No. 1, pp. 158-168, doi: 10.5465/amj.2005.15993166.

[ref037] Ramaswami, A., Dreher, G.F., Bretz, R. and Wiethoff, C. (2010), “Gender, mentoring, and career success: the importance of organizational context”, Personnel Psychology, Vol. 63 No. 2, pp. 385-405, doi: 10.1111/j.1744-6570.2010.01174.x.

[ref038] Roux-Dufort, C. (2009), “The devil lies in details: how crisis builds up within organizations”, Journal of Contingencies and Crisis Management, Vol. 17 No. 1, pp. 4-11, doi: 10.1111/j.1468-5973.2009.00563.x.

[ref039] Rovio-Johansson, A. and Liff, R. (2012), “Sensemaking in a multi professional team”, Journal of Health Organization and Management, Vol. 26 No. 5, pp. 605-620, doi: 10.1108/14777261211256936. 23115907

[ref040] Rubin, H.J. and Rubin, I.S. (2012), Qualitative Interviewing – the Art of Hearing Data, 3rd ed., Sage Publications, California.

[ref041] Scandura, T.A. and Viator, R. (1994), “Mentoring in public accounting firms: an analysis of mentor-protégé relationships, mentorship functions, and protégé turn-over intentions”, Accounting, Organizations and Society, Vol. 19 No. 8, pp. 717-734, doi: 10.1016/0361-3682(94)90031-0.

[ref042] Seibert, S.E., Kraimer, M.L. and Liden, R.C. (2001), “A social capital theory of career success”, Academy of Management Journal, Vol. 44 No. 2, pp. 219-237, doi: 10.5465/3069452.

[ref043] Stallworth, L.H. (2003), “Mentoring, organizational commitment and intentions to leave public accounting”, Managerial Auditing Journal, Vol. 18 No. 5, pp. 405-418, doi: 10.1108/02686900310476873.

[ref044] Straus, S.E., Chatur, F. and Taylor, M. (2009), “Issues in the mentor-mentee relationship in academic medicine: qualitative study”, Academic Medicine, Vol. 84 No. 1, pp. 135-139, doi: 10.1097/acm.0b013e31819301ab. 19116493

[ref045] Strauss, A. and Corbin, J. (1990), Basic of Qualitative Research. Grounded Theory and Procedures and Techniques, Sage, Newbury Park, CA.

[ref046] Swedish Research Council (2017), Good Research Practice, Swedish Research Council, Stockholm.

[ref047] Turban, D.B. and Dougherty, T.W. (1994), “Role of protégé personality in receipt of mentoring and career success”, Academy of Management Journal, Vol. 37 No. 3, pp. 688-702, doi: 10.5465/256706.

[ref048] Turner, B. (1978), Man-Made Disasters, Wykeham Publications, London.

[ref049] Ullrich, L.A., Jordan, R.M., Bannon, J., Stella, J. and Oxenberg, J. (2020), “The mentor match: a new approach to implementing formal mentorship in general surgery residency”, The American Journal of Surgery, Vol. 220 No. 3, pp. 589-592, doi: 10.1016/j.amjsurg.2020.01.011. 31996294

[ref050] Van Manen, M. (1990), Researching Lived Experience. Human Science for an Action Sensitive Pedagogy, The Althouse Press, University of Western Ontario.

[ref051] Weick, K.E. (1988), “Enacted sensemaking in crisis situations”, Journal of Management Studies, Vol. 25 No. 4, pp. 305-317, doi: 10.1111/j.1467-6486.1988.tb00039.x.

[ref052] Weick, K.E., Sutcliffe, K.M. and Obstfeld, D. (2005), “Organizing the process of sensemaking”, Organization Science, Vol. 16 No. 4, pp. 409-421, doi: 10.1287/orsc.1050.0133.

[ref053] Weng, R.H., Huang, C.Y., Tsai, W.C., Chang, L.Y., Lin, S.E. and Lee, M.Y. (2010), “Exploring the impact of mentoring functions on job satisfaction and organizational commitment of new staff nurses”, BMC Health Services Research, Vol. 10 No. 1, 240, doi: 10.1186/1472-6963-10-240. 20712873 PMC2929231

[ref054] Willman, A., Bjuresäter, K. and Nilsson, J. (2020), “Newly graduated nurses' clinical competencies and need for further training in acute care hospitals”, Journal of Clinical Nursing, Vol. 29 Nos 13-14, pp. 2209-2220, doi: 10.1111/jocn.15207. 32043711

[ref055] Yin, R.K. (2016), Qualitative Research from Start to Finish, The Guilford Press, New York.

